# Mesenchymal stem cells: biology and clinical potential in type 1 diabetes therapy

**DOI:** 10.1111/j.1582-4934.2008.00288.x

**Published:** 2008-02-25

**Authors:** Meng Liu, Zhong Chao Han

**Affiliations:** State Key Laboratory of Experimental Hematology, Institute of Hematology, Chinese Academy of Medical Sciences & Peking Union Medical College; National Engineering Research Center of Cell ProductsTianjin, PR China

**Keywords:** mesenchymal stem cells, type 1 diabetes, islet transplantation, insulin-producing cells, cell-based therapy

## Abstract

Mesenchymal stem cells (MSCs) can be derived from adult bone marrow, fat and several foetal tissues. *In vitro*, MSCs have the capacity to differentiate into multiple mesodermal and non-mesodermal cell lineages. Besides, MSCs possess immunosuppressive effects by modulating the immune function of the major cell populations involved in alloantigen recognition and elimination. The intriguing biology of MSCs makes them strong candidates for cell-based therapy against various human diseases. Type 1 diabetes is caused by a cell-mediated autoimmune destruction of pancreatic β-cells. While insulin replacement remains the cornerstone treatment for type 1 diabetes, the transplantation of pancreatic islets of Langerhans provides a cure for this disorder. And yet, islet transplantation is limited by the lack of donor pancreas. Generation of insulin-producing cells (IPCs) from MSCs represents an attractive alternative. On the one hand, MSCs from pancreas, bone marrow, adipose tissue, umbilical cord blood and cord tissue have the potential to differentiate into IPCs by genetic modification and/or defined culture conditions *In vitro*. On the other hand, MSCs are able to serve as a cellular vehicle for the expression of human insulin gene. Moreover, protein transduction technology could offer a novel approach for generating IPCs from stem cells including MSCs. In this review, we first summarize the current knowledge on the biological characterization of MSCs. Next, we consider MSCs as surrogate β-cell source for islet transplantation, and present some basic requirements for these replacement cells. Finally, MSCs-mediated therapeutic neovascularization in type 1 diabetes is discussed.

- Introduction
- Biological characterization of mesenchymal stem cells
  - Isolation and culture of human MSCs
  - Phenotypic properties of MSCs
  - Multi-potent differentiation of MSCs
  - Immunomodulatory effects of MSCs
- Aetiology and current treatment of type 1 diabetes
- Mesenchymal stem cells in type 1 diabetes therapy
  - MSCs with potential to differentiate into insulin-producing cells
  - MSCs as cellular vehicle for insulin gene therapy
  - Induction of IPCs from stem cells by protein transduction technology
  - Minimum requirements for replacement β-cells
  - MSCs for therapeutic neovascularization in type 1 diabetes
- Concluding remarks

## Introduction

Mesenchymal stem cells (MSCs) were first identified by Friedenstein and his colleagues [1], who described bone-forming progenitor cells from rat bone marrow. In addition to postnatal bone marrow, MSCs can also be isolated from adipose tissues, foetal liver, blood, bone marrow, lung, cord blood, placenta and umbilical cord [2–7]. Several lines of evidence have shown that under appropriate environments, MSCs are able to differentiate into mesodermal, endodermal and even ectodermal cells. Another intriguing feature of MSCs is that they escape immune recognition and inhibit immune responses, consequently are called hypoimmunogenic cells. Therefore, MSCs appear to be a very promising tool for regenerative and immunoregulatory cell therapy.

Diabetes mellitus is a devastating metabolic disease, which falls into two categories. Type 1 diabetes results from autoimmune-mediated destruction of β cells in the islets of Langerhans of the pancreas, while type 2 diabetes is due to systemic insulin resistance and reduced insulin secretion by islet β cells. In comparison with conventional or intensive insulin treatment, islet transplantation is the only therapy for type 1 diabetes that achieves an insulin-independent, constant normoglycemic state and avoids hypoglycemic episodes. However, the application of this treatment is restricted by the limited availability of primary human islets from heart-beating donors. Some recent studies indicate that MSCs can differentiate into insulin-producing cells by genetic and/or microenvironmental manipulation *In vitro*. Thus, MSCs provide an alternative β-cell source for islet transplantation.

In this review, we will summarize the major biological features of MSCs, and their possible applications in the treatment of type 1 diabetes.

## Biological characterization of mesenchymal stem cells

### Isolation and culture of human MSCs

Standard conditions for generation of bone marrow derived mesenchymal stromal cultures have been reported [8, 9]. However, the property of plastic adherence itself is not sufficient to obtain purified MSCs, some investigators have tried different methods for isolation of homogenous cell populations [10, 11]. Besides adult bone marrow, researchers in our laboratory have also successfully isolated MSCs from other origins such as foetal lung [12], pancreas, skin, muscle, bone marrow, cord blood and umbilical cord [13]. MSCs in culture have a fibroblastic morphology and adhere to the tissue culture substrate. Under current *in vitro* culture conditions MSCs obtained from young donors can grow to 24–40 population doublings and the proliferative potential of the cells obtained from older donors is more compromised [14]. Afterwards, MSCs enter growth arrest, a phenomenon termed replicative senescence [15]. Replicative senescence is a common characteristic of cultured diploid cells, it is caused by several factors including progressive telomere shortening during continuous subculture *in vitro*[14, 16] due to absence of telomerase activity [17, 18]. Some studies have demonstrated that forced ectopic expression of human telomerase reverse transcriptase (hTERT) in MSCs can dramatically extend their lifespan to >260 population doublings, while maintaining their osteogenic, chondrogenic, adipogenic, neurogenic and stromal differentiation potential [17, 19, 20]. Thus, telomerase activation is a potential strategy for obtaining large number of biologically competent MSCs for clinical application. Unexpectedly, the extensive cell proliferation *in vitro* led to genetic instability and resulted in MSCs transformation [21]. It seems that controllable expression of hTERT gene is very necessary.

### Phenotypic properties of MSCs

Considerable progress has been made towards characterizing the cell surface antigenic profile of human bone marrow-derived MSC populations using fluorescence activated cell sorting (FACS) and magnetic bead-sorting techniques. Nevertheless, to date there is no specific marker or combination of markers that specifically identifies MSCs. Therefore, MSCs have been defined by using a combination of phenotypic markers and functional properties. It is generally agreed that adult human MSCs express Stro-1 [10, 22–23], CD105 (SH2) [24] and CD73 (SH3/4) [25] as well as some cell adhesion molecules including integrins (α1, α2, α3, α5, α6, αV, β1, β3, β4) [26], intercellular adhesion molecule-1, -2 (ICAM-1,-2), vascular cell adhesion molecule-1 (VCAM-1), lymphocyte function-associated antigen 3 (LFA-3), CD72, and activated leucocyte-cell adhesion molecule (ALCAM) [9, 27, 28–30]. They also express human leucocyte antigen (HLA) class I but not class II molecules on cell surface [31]. Additionally, MSCs lack the expression of typical haematopoietic antigens CD45, CD34 and CD14 [27]. (See Table 1 for details).

**Table 1:** Phenotypic properties of mesenchymal stem cells

CD locus	Other names	Detection	References
	Stro-1	Positive	[10, 22, 23]
**CD105**	SH2	Positive	[24]
**CD73**	SH3/4	Positive	[25]
**CD49 a**	α1 integrin	Positive	[26]
**CD49b**	α2 integrin	Positive	[26]
**CD49 c**	α3 integrin	Positive	[26]
**CD49e**	α5 integrin	Positive	[26]
**CD49f**	α6 integrin	Positive	[26]
**CD51**	αV integrin	Positive	[26]
**CD29**	β1 integrin	Positive	[26]
**CD61**	β3 integrin	Positive	[26]
**CD104**	β4 integrin	Positive	[26]
**CD54**	ICAM-1	Positive	[9, 27, 28–30]
**CD102**	ICAM-2	Positive	[9, 27, 28–30]
**CD106**	VCAM-1	Positive	[9, 27, 28–30]
**CD58**	LFA-3	Positive	[9, 27, 28–30]
**CD72**		Positive	[9, 27, 28–30]
**CD166**	ALCAM	Positive	[9, 27, 28–30]
	HLA-I	Positive	[31]
	HLA-II	Negative	[31]
**CD45**		Negative	[27]
**CD34**		Negative	[27]
**CD14**		Negative	[27]

### Multi-potent differentiation of MSCs

A large number of studies demonstrate that bone marrow-derived MSCs from human, canine, rabbit, rat and mouse have the capacity to differentiate into mesenchymal tissues both *In vitro* and *in vivo*, including bone [8, 32], cartilage [33], fat [34, 35], tendon [36, 37], muscle [38, 39] and haematopoietic supporting stroma [35]. In addition, MSCs can differentiate into tissues of ectodermal (*e. g.* neurons) [40] and endodermal (e. g. hepatocytes) origin [41].

Individual colonies derived from single MSC precursors have been reported to be heterogeneous in terms of their multi-lineage differentiation potential [27, 42]. The heterogeneity of adult MSCs could be explained by the notion that in bone marrow, the MSC pool comprises not only putative MSCs, but also subpopulations at different stages of differentiation. Notwithstanding the multi-potentiality of MSCs is a basis for using them to generate different cells and tissues for replacement therapy, the molecular mechanisms that govern MSCs differentiation are incompletely understood. Based on the genetic and genomic information provided by various studies, Baksh *et al.*[43] propose a model for the regulation of adult stem cell differentiation, which incorporates two continuous yet distinct compartments ('stem cell compartment' and ‘ommitment compartment’). The commitment and differentiation of MSCs to specific mature cell types is a tightly and temporally controlled process, involving the activities of various transcription factors, cytokines, growth factors, and extracellular matrix molecules. Global gene expression profiling using DNA microarray technology has already been used successfully to identify genes that regulate osteogenic, adipogenic and chondrogenic differentiation of MSCs [44, 45], which has greatly facilitated our effort to elucidate the mechanism controlling adult stem cell differentiation. The traditional view of linear hierarchical progression of stem cells from one differentiation stage to the next during their phenotypic determination has been challenged by the recent findings [46–48]. Using an *In vitro* differentiation strategy, Song *et al.*[49] showed that MSC-derived, fully differentiated osteoblasts, adipocytes and chondrocytes can switch their phenotypes to other mesenchymal lineages in response to specific extracellular stimuli. Taken together, it could be concluded that both pre-committed progenitor cells and terminally differentiated cells retain the multi-potency, and that their plasticity can be preserved during differentiation and be required under defined, appropriate microenvironmental circumstances.

### Immunomodulatory effects of MSCs

MSCs have been shown to suppress immune reactions both *In vitro* and *in vivo* in a non-MHC restricted manner [50]. These stem cells are considered to be hypoimmunogenic, displaying low expression levels of HLA class I and no expression of costimulatory molecules, such as B7–1 (CD80), B7–2 (CD86) and CD40 [26, 51, 52]. *In vitro*, MSCs are able to suppress T lymphocyte proliferation induced by alloantigens [50, 51, 53, 54], mitogens [50, 55–58], as well as activation of T cells by CD3 and CD28 antibodies [51, 59, 60]. Suppression of T cell proliferation by MSCs has no immunological restriction, similar suppressive effects being observed with cells that were autologous or allogeneic to the responder cells [50, 52, 53, 61]. Another level at which MSCs modulate immune responses is through the induction of regulatory T cells. MSCs have been reported to induce formation of CD8^+^ regulatory T cells that were responsible for inhibition of allogeneic lymphocyte proliferation [58]. Furthermore, an increase in the population of CD4^+^ CD25^+^ regulatory T cells has been demonstrated in mitogen-stimulated peripheral blood mononuclear cell (PBMCs) cultures in the presence of MSCs [60–61]. However, depletion of CD4^+^ CD25^+^ regulatory T cells had no effect on the suppression of T cell proliferation by MSCs [59]. Apart from naive and memory T cells [59], MSCs can also inhibit several functions of B cells [62], natural killer cells [63, 64] and monocyte-derived dentritic cells [65, 66]. Although the exact mechanism underlying the immunosuppressive effects of MSCs has not been fully clarified, most studies supported that soluble factors are involved. These factors include transforming growth factor (TGF)-β 1 [56, 67], hepatocyte growth factor (HGF) [56, 63], prostaglandin E2 (PGE2) [60, 63] and indoleamine 2,3-dioxygenase (IDO) [68–70]. Additionally, it is well-established that IFN-γ plays an important role in the enhancement of MSCs' suppressive activity [31, 60, 68, 69].

The immunomodulatory capacity of MSCs has also been evaluated *in vivo*. First, intravenous administration of MSCs derived from BM of baboons prolonged the survival of allogeneic skin grafts [53]. Subsequently, murine MSCs have been demonstrated to prevent experimental autoimmune encephalomyelitis (EAE) in mice [71]. In phase I studies, Lazarus *et al.*[72, 73] estimated the feasibility of transplanting autologous or allogeneic MSCs to improve engraftment of HSCs, as well as to reduce graft-versus-host disease (GVHD). Another clinical trial also displayed that third party haplo-identical (mother-derived) MSCs can be safely infused to treat severe acute GVHD that is refractory to conventional immunosuppressive therapy [74]. In contrast, infusion of MSCs had no beneficial effects on collagen-induced arthritis (CIA) as tested in a murine model of rheumatoid arthritis (RA) [75]. Grinnemo *et al.*[76] observed that after transplantation of human MSCs into experimentally induced ischaemic rat myocardium, MSCs induced significant lymphocyte proliferation in PBMC cultures of immunized rats. Moreover, there was prominent infiltration of macrophages in the area of injection in immunocompetent rats. Therefore, though MSCs have been shown to be transplantable across allogeneic barriers, xenogeneic transplant rejection may occur.

### Aetiology and current treatment of type 1 diabetes

In the year 2000, 150 million people worldwide were found to be affected by diabetes mellitus, and this number is considered to double in 2025 [77]. Type 1 diabetes is characterized by the selective destruction of pancreatic β-cells caused by an autoimmune attack, and it accounts for 5–10% of all causes of diabetes mellitus. Autoimmune destruction of β-cells is due to multiple genetic predispositions and is also related to environmental factors that are still poorly defined [78]. When clinical symptoms are observed the autoimmune process is markedly advanced. It is reported that 60–80% of the β-cell mass have been destroyed at the time of diagnosis [79].

Since 1920s, insulin therapy has changed diabetes from a rapidly fatal disease to a chronic disease associated with significant secondary complications, such as renal failure, cardiovascular disease, retinopathy and neuropathy. It is now well-established that the risk of diabetic complications is dependent on the degree of glycaemic control in diabetic patients. Long-term studies strongly suggest that tight control of blood glucose achieved by conventional or intensive insulin treatment, self blood glucose monitoring, and patient education can significantly prevent the development and retard the progression of chronic complications of this disease [80–82]. While aggressive insulin therapy that maintains glucose levels near the normal range reduces the risk of secondary complications, patients often find such control difficult to achieve and suffer an increased risk of hypoglycaemia [83]. This is caused by the fact that external insulin injection can not mimic the physiological control that pancreatic β cell-derived insulin secretion exerts on the body's glycaemia. By contrast, replacement of a patient's islets of Langerhans either by whole pancreas transplantation or by isolated islet transplantation is the only treatment of type 1 diabetes that achieves an insulin-independent, constant normoglycaemic state and avoids hypoglycaemic episodes [84, 85]. Nonetheless, due to shortage of organs and lifelong immunosuppression this therapy can be offered to a very limited number of patients. What is now required is an essentially infinite supply of a physiologically competent substitute for primary human pancreatic islets, and generation of insulin-producing cells from stem cells represents an attractive alternative [86].

## Mesenchymal stem cells in type 1 diabetes therapy

### MSCs with potential to differentiate into insulin-producing cells

Among adult stem cells, MSCs appear to have a particular developmental plasticity *ex vivo* that include their ability to adopt a pancreatic endocrine phenotype. It has been demonstrated that MSCs residing in various tissues and organs are able to differentiate into functional insulin-producing cells, such as MSCs from pancreas, bone marrow, adipose tissue, cord blood and cord tissue. This will help to meet the demand of β cells for islet transplantation, and the goal of a permanent cure for type 1 diabetes will be realized.

The mature pancreas has two functional compartments: the exocrine portion (99%), including acinar and duct cells, and the endocrine portion (1%), including the islets of Langerhans. Islets are composed of four cell types that synthesize and secrete distinct peptidic hormones: β-cells (insulin), α-cells (glucagon), δ-cells (somatostatin) and PP-cells (pancreatic polypeptide). It has been described that adult rat and human islets of Langerhans contain nestin-positive progenitor cells, which can be differentiated into insulin-expressing cells *ex vivo*[87]. In another study, Ramiya *et al.*[88] displayed how pluripotent stem cells isolated from the pancreatic ducts of adult pre-diabetic non-obese diabetic (NOD) mice differentiate to form glucose-responsive islets that can reverse insulin-dependent type 1 diabetes after being implanted into diabetic NOD mice. Simultaneously, duct tissue from human pancreas was expanded and directed to differentiate into functional islet tissue *In vitro*[89]. Then, Bonner-Weir *et al.*[90] considered that ductal epithelial cells are likely to be the pancreatic progenitors which can add new β cells by the process of neogenesis. The clonal identification of multi-potent precursor cells from adult mouse pancreas that generate endocrine β-like cells were also performed [91]. Recently, several studies have indicated that MSCs are likely to exist within pancreatic duct and islet. Zhang *et al.*[92] showed that nestin-positive cells isolated from human foetal pancreas possess the characteristics of pancreatic progenitor cells since they have highly proliferative potential and the capability of differentiation into insulin-producing cells *In vitro*. Huang et al. [93] further proved that after differentiation the islet-like cell clusters (ICCs) displayed the ability to reverse hyperglycaemia in diabetic mice. Additionally, these nestin-positive pancreatic progenitor cells share many phenotypic markers with MSCs derived from bone marrow [92]. In agreement with these findings, another group [94] successfully isolated pancreatic stem cells from adult human pancreatic duct, these cells not only express nestin and pdx-1 but also exhibit the identical markers of MSCs. Moreover, Seeberger and his colleagues [95] reported the expansion of MSCs from adult human pancreatic ductal epithelium. In addition to expression of the same surface antigens as MSCs from human bone marrow, adipose and umbilical cord blood [11, 96, 97], they demonstrated that pancreatic MSCs could be differentiated into mesodermal cells including osteocytes, adipocytes and chondrocytes. Their preliminary data also suggest that these cells have the potential to derive β-cells. An earlier study has established that fibroblast-like precursor cells derived from adult human islets are generated by epithelial-to-mes-enchymal transition (EMT) [98]. However, in a recent paper, researchers verified that EMT does not underlie the appearance of fibroblast-like cells in mouse islet cultures, but that fibroblast-like cells appear to represent MSC-like cells akin to MSCs isolated from bone marrow [99]. More recently, it has been revealed that human islet-derived precursor cells (hIPCs), which do not express the insulin gene, nonetheless exhibit transcriptionally active epigenetic marks. These findings in hIPCs may be an indication of the ‘committed state’ of hIPCs as endocrine pancreas precursor cells [100]. In conclusion, MSCs in human pancreas could serve as a competent candidate for generating insulin-producing cells.

Bone marrow is an important source of easily accessible adult stem cells, and bone marrow transplantation (BMT) is considered to be effective for the treatment of autoimmune type 1 diabetes. However, there is a great debate on the issue of the fate of transplanted bone marrow stem cells. Ianus *et al.*[101] showed that mouse bone marrow-derived cells can differentiate into pancreatic endocrine β cells with glucose-dependent and incretin-enhanced insulin secretion when transplanted into lethally irradiated mice. By using a CRE-LoxP system, the authors also ruled out cell fusion events. Many controversial observations still exist. Hess *et al.*[102] reported that transplantation of c-kit positive mouse bone marrow-derived stem cells initiated endogenous pancreatic regeneration and improved blood glucose level in streptozocin (STZ)-induced diabetic mice *via* enhanced endothelial proliferation by donor cells. In a similar study, Lee *et al.*[103] demonstrated that transplanted MSCs from human bone marrow lowered blood glucose levels in diabetic immunodeficient mice by promoting repair of mouse pancreatic islets. Furthermore, independent studies by Choi *et al.*[104], Lechner *et al.*[105] and Taneera *et al.*[106] showed little evidence for significant transdifferentiation of bone marrow cells (BMCs) into pancreatic β cells, even in pancreatic injury models of mice. Lately, cotransplantation of syngeneic BMCs and syngeneic or allogeneic MSCs into diabetic mice resulted in rapid recovery of blood glucose and serum insulin levels accompanied with efficient tissue regeneration. Researchers suggested that two aspects operate parallelly and synergistically in this model. First, BMCs and MSCs induce the regeneration of recipient derived pancreatic insulin-secreting cells. Second, MSCs inhibit T cell-mediated immune responses against newly formed β-cells. Their work offers a novel potential therapeutic protocol for type 1 diabetes [107]. On the other hand, recent studies illustrated that when cultured *In vitro*, bone marrow derived-cells obtained from mice [108] and rats [109] could be differentiated into insulin-producing cells. Multi-potent adult progenitor cells (MAPCs) or MSCs within bone marrow are intriguing candidates that can give rise to insulin-positive cells. In 2002, Jiang *et al.*[110] proposed the existence of pluripotent MSCs derived from adult marrow. Chen et al. [111] and Wu *et al.*[112] isolated MSCs from rat bone marrow, and successfully induced their differentiation into islet-like cells. Moreover, transplantation of these islet-like cells could alleviate the hypergly-caemia in diabetic rats. Subsequently, a group of researchers [113] proved that treatment of rat pancreatic extract can differentiate rat marrow mesenchymal cells into insulin-producing cells *In vitro*. In another study, Moriscot et al. [114] indicated that human bone marrow MSCs are able to differentiate into insulin-expressing cells by infection with adenoviruses coding for several transcription factors of the β-cell developmental pathway and coculture with islet tissue or islet-conditioned medium. Recently, two studies [115, 116] have presented evidence that pancreatic duodenal homeobox-1 (PDX-1) gene-modified human bone marrow-derived MSCs can be induced to differentiate into functional insulin-producing cells. In addition, Sun *et al.*[117] demonstrated that bone marrow-derived MSCs from diabetic patients can differentiate into IPCs under appropriate conditions *In vitro*. Their results provide the direct evidence for the feasibility of using patient's own BM-MSCs as a source of IPCs for beta-cell replacement therapy.

MSCs from human bone marrow and adipose tissue represent very similar cell populations with comparable phenotypes [2, 96, 118–119]. Thus, MSCs with the potential to adopt a pancreatic endocrine phenotype could also exist in human adipose tissue. Timper *et al.*[120] isolated human adipose tissue-derived MSCs and expanded them in basic fibroblast growth factor (bFGF) containing culture medium. Proliferating MSCs expressed the stem cell markers nestin, ABCG2, SCF, Thy-1 as well as the pancreatic endocrine transcription factor Isl-1 mRNA. When subjected to defined differentiation medium, a down-regulation of ABCG2 and an up-regulation of transcription factors Isl-1, Ipf-1 and Ngn3 were observed together with induction of the islet genes insulin, glucagon and somatostatin. Consequently, adipose tissue-derived MSCs could be an alternative source of pancreatic β-cells.

Human umbilical cord blood (HUCB) is another source of stem cells with the potential to develop into insulin-producing cells. A few *in vivo* studies give support to this point. In one study [121], transplantation of HUCB cells resulted in the improvement of blood glucose levels and survival rate in type 2 diabetic mice. Furthermore, a regression of glomerular hypertrophy and tubular dilatation, common complications attributed to diabetes, was observed in HUCB-treated mice. In another study [122], transplantation of HUCB cells into type 1 diabetic mice led to a dose-dependent reduction in blood glucose levels and the degree of autoimmune insulitis. A recent report [123] has focused on the *in vivo* capacity of HUCB-derived cells to generate insulin-producing cells. Following transplantation of HUCB cells into NOD/SCID/β2m^null^ mice, IPCs of human origin were found in recipient pancreatic islets. Double FISH analysis using species-specific probes further indicated that HUCB cells can give rise to insulin-producing cells by fusion-dependent and -independent mechanisms. The number of HUCB cells that transdifferentiated and the rate of such an event are critical aspects. The proportion of HUCB-derived insulin-producing cells per total number of islet cells [123] was less than in the case of BM-derived insulin-producing cells [101]. However, under diabetic conditions, the demand for the neogenesis of insulin-producing cells might increase and the rate of HUCB cell differentiation could become higher in order to compensate for the regeneration of β-cell mass. On the other hand, the stem cell type in HUCB responsible for generation of insulin-producing cells remains unclear. Since MSCs have been identified in the cord blood [124] and HUCB-derived USSC (unrestricted somatic stem cell) share most of the cell markers and properties with MAPCs [125], it should be considered that MSCs may take part in the differentiation of HUCB cells towards a β-cell phenotype. In addition to HUCB, the Wharton's jelly of the human umbilical cord is rich in mesenchymal stem cells (UC-MSCs) that fulfil the criteria for MSCs. Recently, Chao *et al.*[126] successfully differentiated UC-MSCs into mature ICCs, and these ICCs possess insulin-producing ability *In vitro* and *in vivo*. Moreover, they indicated that UC-MSCs seem to be the preferential source of stem cells to convert into IPCs, because of the large potential donor pool, its rapid availability, no risk of discomfort for the donor, and low risk of rejection.

### MSCs as cellular vehicle for insulin gene therapy

MSCs are a promising target population for cell-based gene therapy against a variety of different diseases [127]. The apparently high self-renewal potential makes them strong candidates for delivering genes and restoring function of organs and tissues. The ability to genetically modify MSCs provides a means for durable expression of therapeutic genes. Following the development of better assays for stem cells and improvements in vector biology, gene transfer efficiencies into MSCs have increased prominently. To assess the capacity of MSCs to produce heterologous proteins, many transgenes were expressed in MSCs *In vitro*. The proteins included coagulation factors VIII [128], IX [129], IL-3 [130], human growth hormone [131], human ery-thropoietin (hEPO) [132] and so on. As a result, MSCs could act as platforms for recombinant protein production *in vivo* to treat acquired and inherited disorders. As far as type 1 diabetes is concerned, insulin gene therapy using MSCs is an alternative treatment.

Human insulin gene is located on chromosome 11p15.5 [133]. Insulin synthesis and release from islet β-cells is complex and tightly regulated. Glucose affects insulin at all levels, including transcription, translation and release. Mature insulin results from a processing pathway which starts at the rough endoplasmic reticulum and ends at the Golgi apparatus. Translation of insulin mRNA yields pre-proinsulin, which is sequentially cleaved by endoproteinases PC1 and PC2/PC3 to give pro-insulin first and mature insulin plus C-peptide second. In the secretory granule, six insulin molecules are coordinated by a Zn atom, which is demonstrated under microscopy by dithizone staining. Some researchers have begun to set foot in the field of MSCs-based insulin gene therapy for type 1 diabetes. In one study [134], human bone marrow MSCs transduced with adeno-associated virus (AAV) containing furin-cleavable human preproinsulin gene produce increased amount of insulin and C-peptide compared to the control group. In another study [135], retrovirus vector pLNCX was used to transfer the human insulin gene into human BM-MSCs. The transfected MSCs expressed the insulin gene and stably secreted insulin into culture media. More recently, Xu *et al.*[136] showed that experimental diabetes in mice could be relieved effectively for up to 6 weeks by intrahepatic transplantation of bone marrow-derived murine MSCs infected with the recombinant retro-virus-carrying human insulin gene. However, implantation of engineered cells using diabetic animal models and evaluation of therapeutic effect should be performed with more tests of efficacy and safety of engineered human MSCs as surrogate β-cells in further study. In addition, other researchers [137] are working with a modified herpes I virus as a vector for the human insulin gene. The theoretical advantages of the herpes I virus are: (*i*) the large capacity to accommodate a construct; (*ii*) the ability of the virus to infect primary and second cell lines *In vitro*; (*iii*) although the virus enters the nucleus it does not integrate with the host DNA and is therefore not likely to unmask oncogenes, it functions separate to the host DNA as an episome; (*iv*) most patients have already had contacts with the herpes I virus, which normally resides in a quiescent state in neurological tissue; (*v*). immune reaction against the virus is relatively mild; (*vi*) established antiviral treatment against the herpes virus is available. In consequence, the modified herpes I virus could serve as a new vector for human insulin gene delivery into MSCs. (Table 2)

**Table 2:** Cell-based treatment protocols in experimental diabetes models

Study	Cell source for transplantation	Therapeutic effects in diabetic animal models
**Ramiya *et al.*[88]**	Islets generated from mouse pancreatic stem cells	Insulin-independent, blood glucose levels return to near-normal levels
**Huang *et al.*[93]**	ICCs derived from NIPs residing in human foetal pan-creas	Reverse hyperglycaemia
**Hess *et al.*[102]**	mouse c-kit^+^ BM-derived cells	Reduce hyperglycaemia, accompanied by a proliferation of recipient pancreatic cells
**Lee *et al.*[103]**	Human BM-MSCs	Lower blood glucose levels, promote repair of pancreatic islets and renal glomeruli
**Urbán *et al.*[107]**	Mouse syngeneic BMCs and syngeneic or allogeneic MSCs	Rapid recovery of blood glucose and serum insulin levels accompanied with efficient pancreatic tissue regeneration
**Tang *et al.*[108]**	IPCs obtained from mouse bone marrow	Reverse hyperglycaemia, improve metabolic profiles
**Oh *et al.*[109]**	IPCs transdifferentiated from rat BMCs	Lower blood glucose levels, maintain comparatively normal glucose levels
**Chen *et al.*[111] and Wu *et al.*[112]**	Islet-like cells differentiated from rat marrow MSCs	Lower glucose levels
**Li *et al.*[115] and Karnieli *et al.*[116]**	IPCs generated from PDX-1 gene-modified human BM-MSCs	Reduction of hyperglycaemia
**Ende *et al.*[121, 122]**	HUCB mononuclear cells	Improve blood glucose levels, survival rate, glomerular hypertrophy, tubular dilatation and insulitis
**Chao *et al.*[126]**	ICCs derived from human UC-MSCs	Alleviate hyperglycaemia and glucose intolerance sig-nificantly
**Xu *et al.*[136]**	Mouse BM-MSCs infected with recombinant retrovirus-carrying human insulin gene	Improvement of body weight, blood glucose and serum insulin levels

Abbreviations: ICCs, islet-like cell clusters; NIPs, nestin-positive islet-derived progenitor cells; MSCs, mesenchymal stem cells; BMCs, bone marrow cells; IPCs, insulin-producing cells; PDX-1, pancreatic duodenal homeobox-1; HUCB, human umbilical cord blood; UC, umbilical cord.

### Induction of IPCs from stem cells by protein transduction technology

New technology, known as protein transduction technology, has been recently developed. A variety of peptides, known as protein transduction domains (PTDs) or cell-penetrating peptides (CPPs), have been characterized for their ability to translocate into live cells. Proteins and peptides can be directly internalized into cells when synthesized as recombinant fusion proteins or covalently cross-linked to PTDs. There are numerous examples of biologically active full-length proteins and peptides that have been delivered to cells both *In vitro* and *in vivo*. The most commonly studied PTDs are homeodomain transcription factors such as Antennapedia (Antp), HSV type 1 protein VP22 and HIV-1 transactivator TAT protein. The mechanism of PTD-mediated protein transduction is mainly via endocytosis followed by passage from the vesicle into the cytoplasm [138].

It has been suggested that protein transduction technology is useful for the treatment of diabetes, because this technology facilitates the differentiation of stem cells into insulin-producing cells. First, PDX-1 protein and BETA2/NeuroD protein, two pancreatic endocrine transcription factors, both have a PTD sequence in their structure. Noguchi *et al.* demonstrated that PDX-1 [139] or BETA2/NeuroD [140] protein induced insulin expression in pancreatic ductal progenitor cells. Similarly, Domínguez-Bendala *et al.*[141] showed that TAT-mediated neurogenin 3 (ngn3) protein transduction stimulated pancreatic endocrine differentiation *In vitro*. In another research, Gräslund's group [142] reported that the third helix of the homeodomain of transcription factor Isl-1 internalized into cells. Thus, delivery of exogenous transcription factors (PDX-1, BETA2/NeuroD, ngn3, Isl-1, etc.) by protein transduction technology could be a novel strategy for generating IPCs from stem/progenitor cells without requiring gene transfer technology. We propose MSCs as strong candidate stem cells for this new approach.

### Minimum requirements for replacement β-cells

As mentioned above, insulin-producing cells generated either by transdifferentiation of MSCs or by delivery of insulin gene into MSCs are able to act as replacement β-cells for the transplantation therapy of type 1 diabetes. These MSCs-derived IPCs may solve the donor shortage issue for islet cell transplantation and provide a cure for this disease. Nevertheless, any substitute for primary islets of Langerhans will require some minimum essential properties. The basic requirements for surrogate β-cells are described as follows [143].

First, to make any significant therapeutic impact vast numbers of replacement β-cells will be required. Current transplantation protocols use up to 1 × 10^6^ primary human islets per recipient, equivalent to approximately 2–4 × 10^9^β-cells. As a result, the ability of MSCs to replicate and to differentiate toward pancreatic endocrine phenotype makes them attractive candidates for producing replacement β-cells. Secondly, the replacement cells must have the ability to synthesize, store and release insulin in response to changes in the ambient glycaemia. Understanding β-cell function at the molecular level will likely facilitate to manufacture physiologically competent insulin-producing cells from MSCs. Thirdly, the proliferative capacity of the replacement cells must be tightly controlled to avoid the development of hyperinsulinemic hypogly-caemia as the β-cell mass expands *in vivo*. Excluding proliferative cells from the transplant material will help to overcome this problem. In the case of insulin gene transferred MSCs, the possibility of tumour formation has to be considered. Finally, the transplanted cells must avoid destruction by the recipient's immune system. Two major mechanisms are involved in the immune attack against replacement β-cells, one is transplant rejection and the other is recurrence of autoimmunity. In addition to appropriate immunosuppressive treatment, autologous transplantation of MSCs-derived IPCs will circumvent the immune rejection dilemma. On the other hand, Burt *et al.*[144] indicated that HSC transplantation may re-introduce tolerance to islet cells in type 1 diabetics. Thus, cotransplantation of MSCs-derived IPCs and HSC from the same donor (autologous or allogeneic) could evade the risks of recurring autoimmunity. Furthermore, the pathways of β-cell differentiation *In vitro* may differ significantly from those *in vivo*[145], and it is also possible that current *In vitro* differentiation protocols do not generate β-cells, but cells that have somephenotypic and functional similarity to authentic β-cells. Since IPCs generated from MSCs are developmentally and immunologically distinct from primary β-cells, they may escape the recipient's autoimmune assault.

### MSCs for therapeutic neovascularization in type 1 diabetes

It has been demonstrated that endothelial progenitor cells (EPCs) are responsible for postnatal vasculogenesis in physiological and pathological neovascularization [146]. Ischaemia and tissue injury are potent stimuli for neovascularization. We have reported that autologous transplantation of granulocyte colony-stimulating factor-mobilized peripheral blood mononuclear cells (M-PBMSCs) improves critical limb ischaemia (CLI) in diabetes [147]. Further investigation indicated that local transplantation of M-PBMNCs achieved therapeutic neovascularization via supply of abundant angioblasts (EPCs) and angiogenic factors [148]. However, EPCs in type 1 diabetic patients are dysfunctional, and their dysfunction may contribute to the pathogenesis of vascular complications in type 1 diabetes [149]. Our group also proved that M-PBMNCs from diabetic patients augment neovascularization in ischaemic limbs but with impaired capability [150]. Clinically, allogenic transplantation of normal M-PBMNCs may be more effective, but such transplanted cells are likely to encounter immune rejection. Therefore, autologous transplantation of diabetic M-PBMNCs is still a good-albeit compromised and not perfect-approach for CLI in diabetes. On the other hand, the pancreatic islets of Langerhans are well vascularized throughout life. Signals from the endothelium may play a role in postnatal islet cell proliferation and neogenesis. Mathews *et al.*[151] provided evidence that transplanted bone marrow-derived EPCs are recruited to the pancreas in response to STZ-induced islet injury and that EPC-mediated neo-vascularization of the pancreas could in principle facilitate the recovery of non-terminally injured β-cells. Neovascularization of the pancreas is likely to be an adaptive response to β-cell injury in type 1 diabetes.

**Fig. 1:**
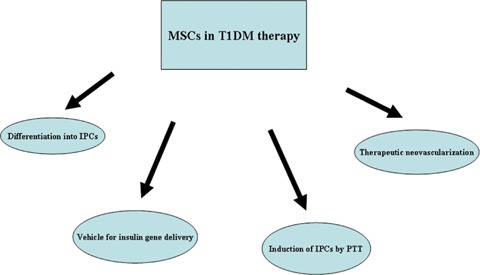
Mesenchymal stem cells in the treatment of type 1 diabetes. The clinical potentials of mesenchymal stem cells (MSCs) in type 1 diabetes therapy are illustrated. Abbreviations: T1DM, type 1 diabetes mellitus; IPCs, insulin-producing cells; PTT, protein transduction technology.

MSCs have been shown to promote angiogenesis both *in vivo*[152] and *In vitro*[153]. Yet the underlying mechanism of this action remains elusive. Oswald *et al.*[154] showed the differentiation of expanded adult human BM-MSCs into cells with pheno-typic and functional features of endothelial cells. However, Kinnaird *et al.*[155] demonstrated that MSCs secrete a wide array of arteriogenic cytokines and they contribute to collateral re-mod-elling in ischaemic limb via paracrine mechanisms. Recently, another two studies suggest that BM-MSCs enhance angiogenesis in wounds of diabetic mice through paracrine effects [156, 157]. An increasing bulk of evidence supports that release of angiogenic factors rather than endothelial transdifferentiation is accountable for MSCs-mediated strengthened angiogenesis. MSCs express genes encoding a broad spectrum of arteriogenic/angiogenic cytokines including vascular endothelial growth factor (VEGF), fibroblast growth factor (FGF), Angiopoetin-1 (Ang-1), matrix met-alloproteinase (MMPs), transforming growth factor-β (TGF-β) and so on [158]. For example, MSCs have been reported to generate sufficient quantities of VEGF to enhance survival and differentiation of endothelial cells [159]. In addition to stimulating the in situ proliferation of endothelial cells, VEGF has also been shown to promote neovascularization by mobilizing bone marrow-derived EPCs [160]. Thus, transplanted MSCs may initiate angiogenesis in diabetic ischaemic limbs or injured pancreas by producing angiogenic factors. Neovascularization will become a new direction for the application of MCSs in type 1 diabetes therapy. (Fig. 1)

## Concluding remarks

In the past few years, there has been dramatic progress in our understanding of the biology of MSCs. Data in the literature concerning cell expansion, phenotypic characterization of MSCs as well as their multi-potency and immunomodulatory properties, are vast and sometimes contradictory. Although the precise identity of MSCs remains a challenge, this has not hampered the beginning of considerable investigation aiming at their potential clinical applications. It is generally accepted that type 1 diabetes is now curable by islet transplantation therapy, and MSCs offer a starting material for generating the large numbers of surrogate β-cells required. The most difficult and yet unsolved issue are how to manufacture physiologically functional insulin-producing cells from MSCs. Moreover, the angiogenic effect of MSCs could also be utilized for diabetes treatment. In conclusion, the prospect of MSCs in treating type 1 diabetes seems to be very promising. However, we should realize that much work needs to be done before pushing the MSC-based therapy from bench to bedside.
